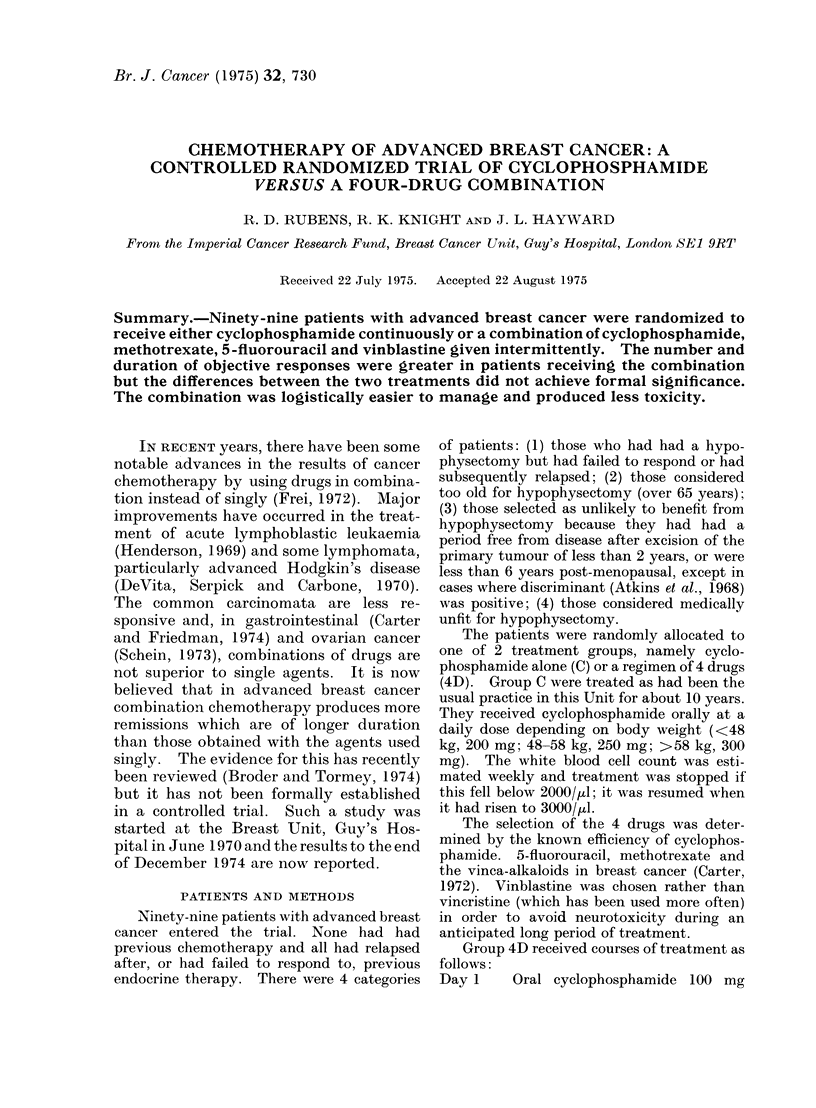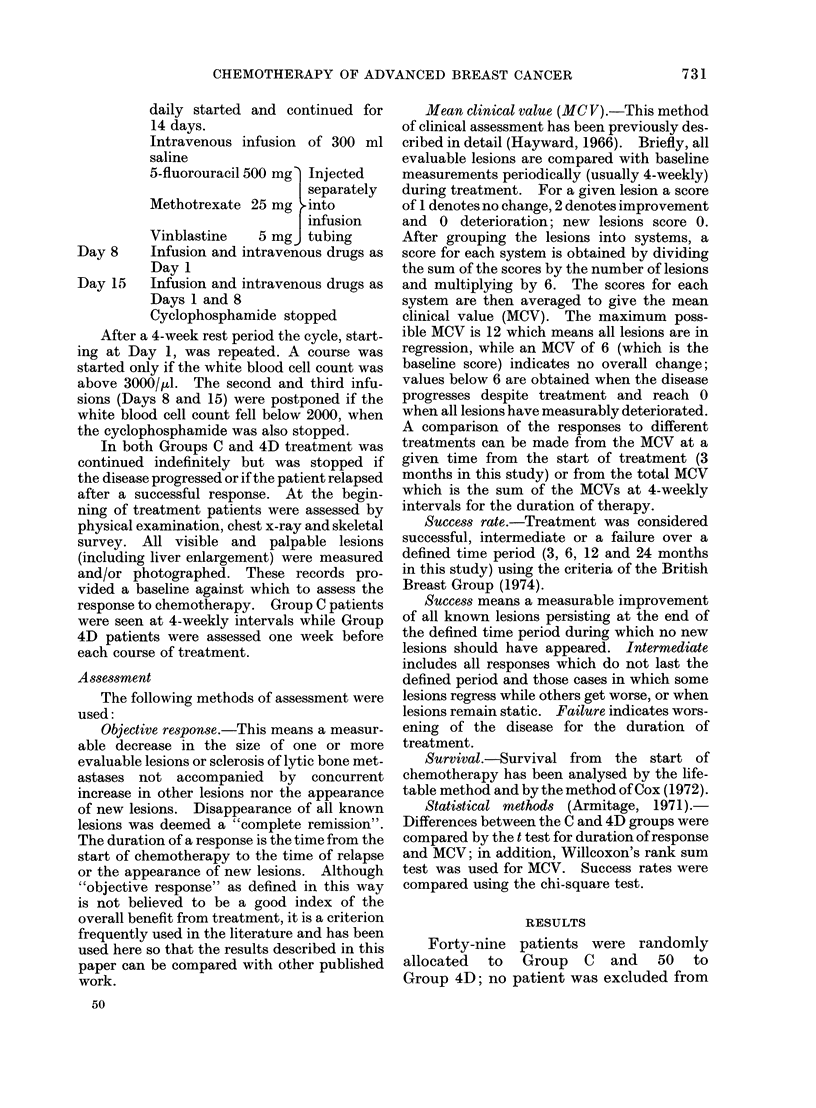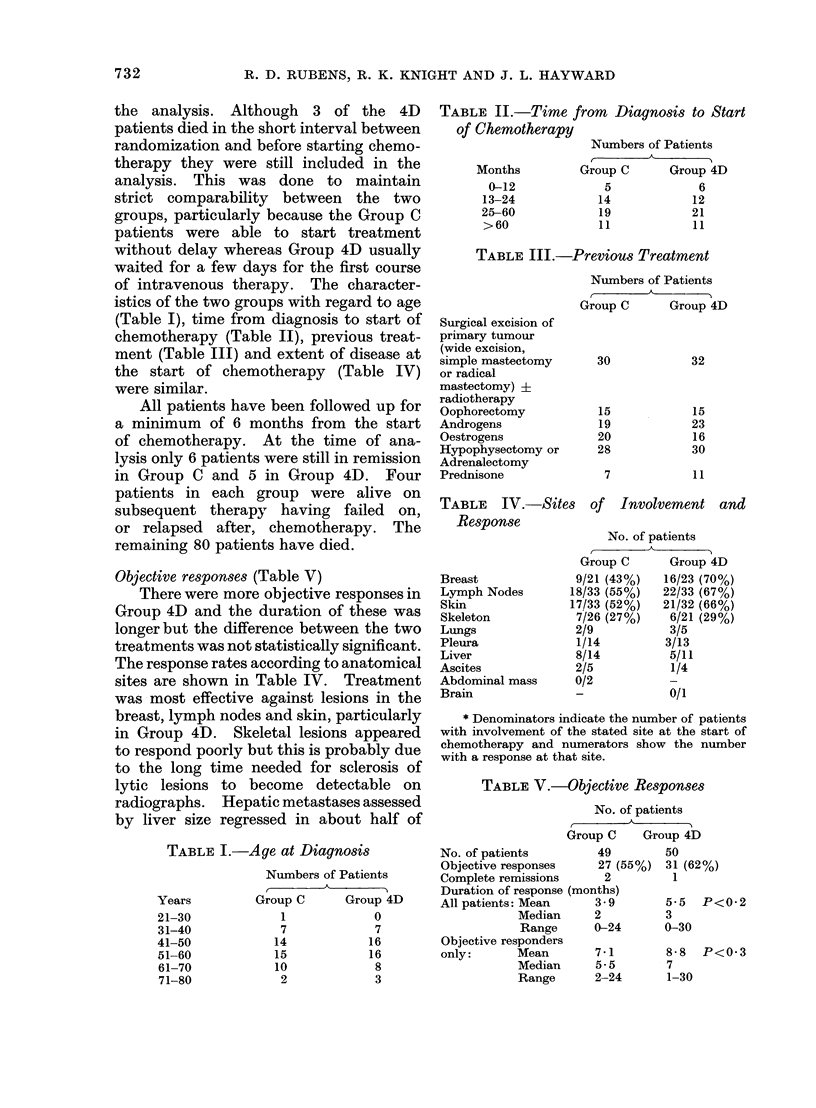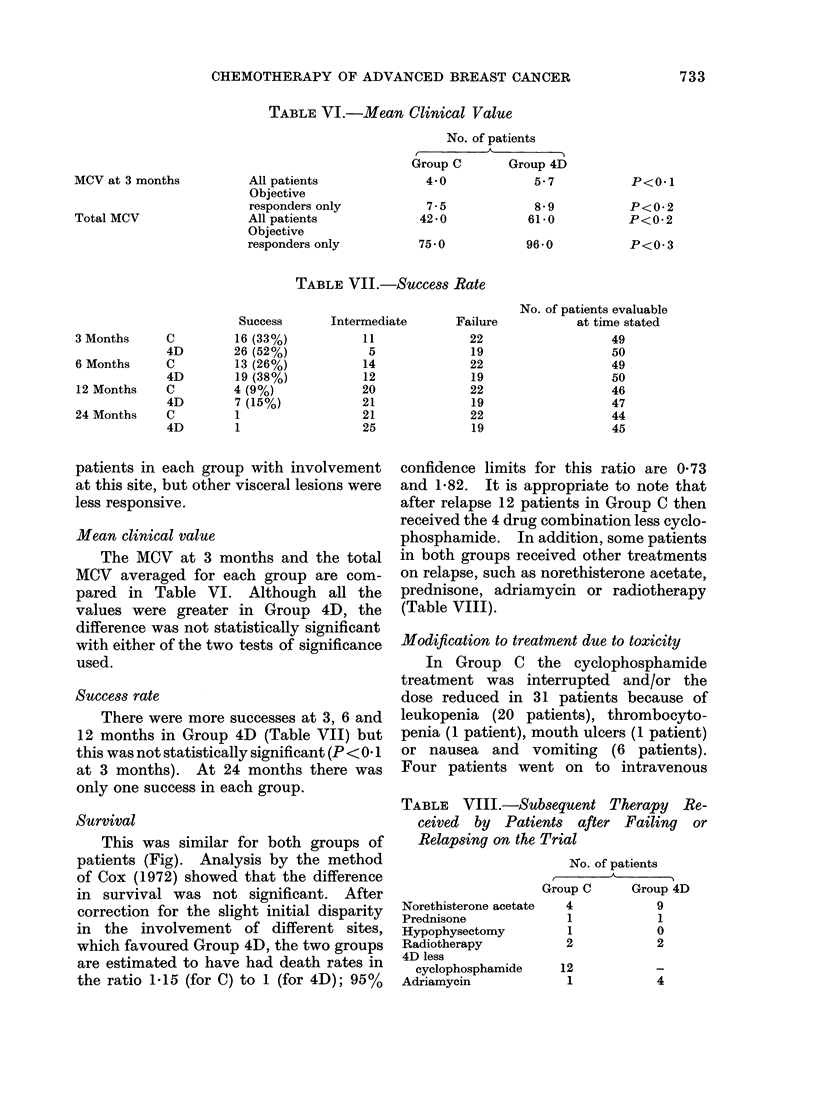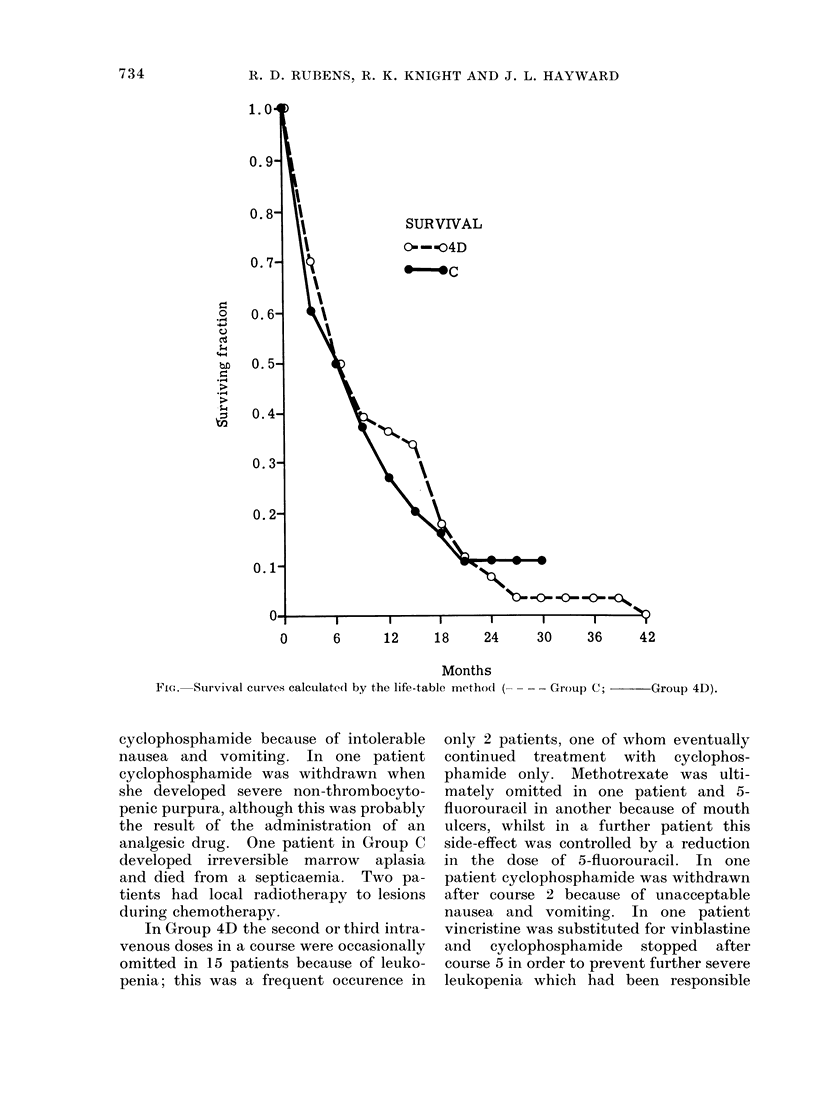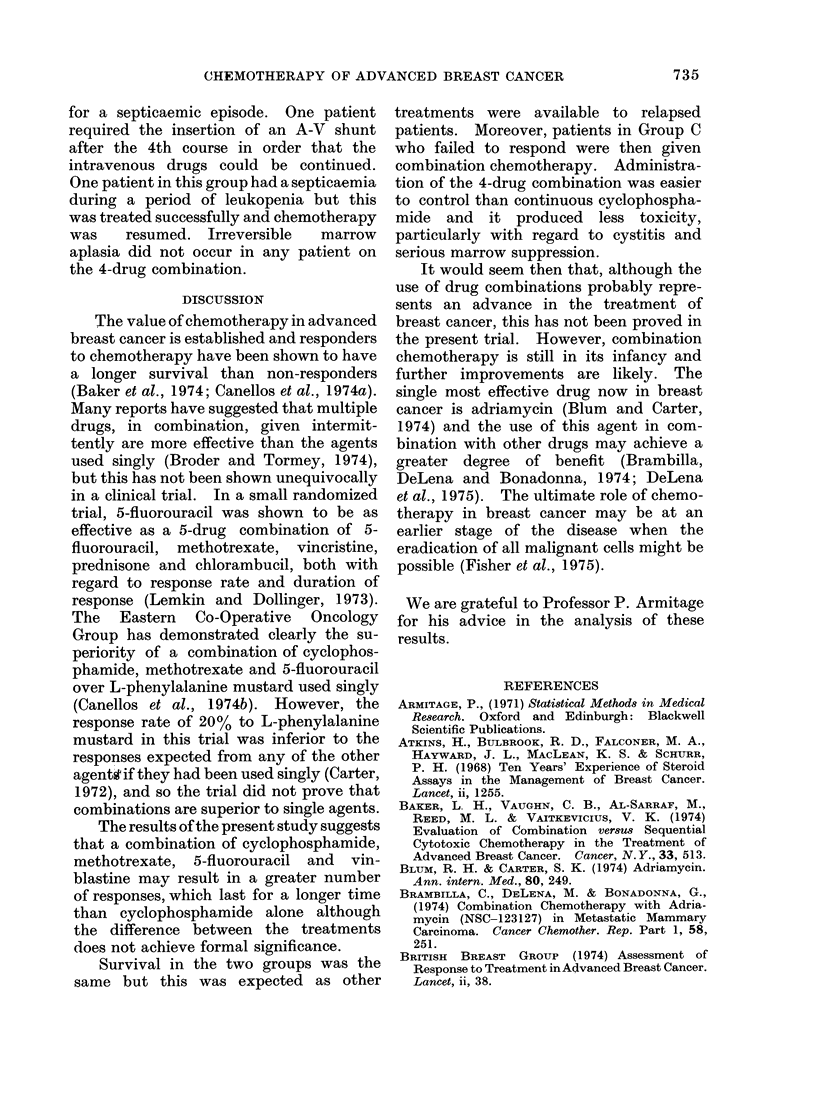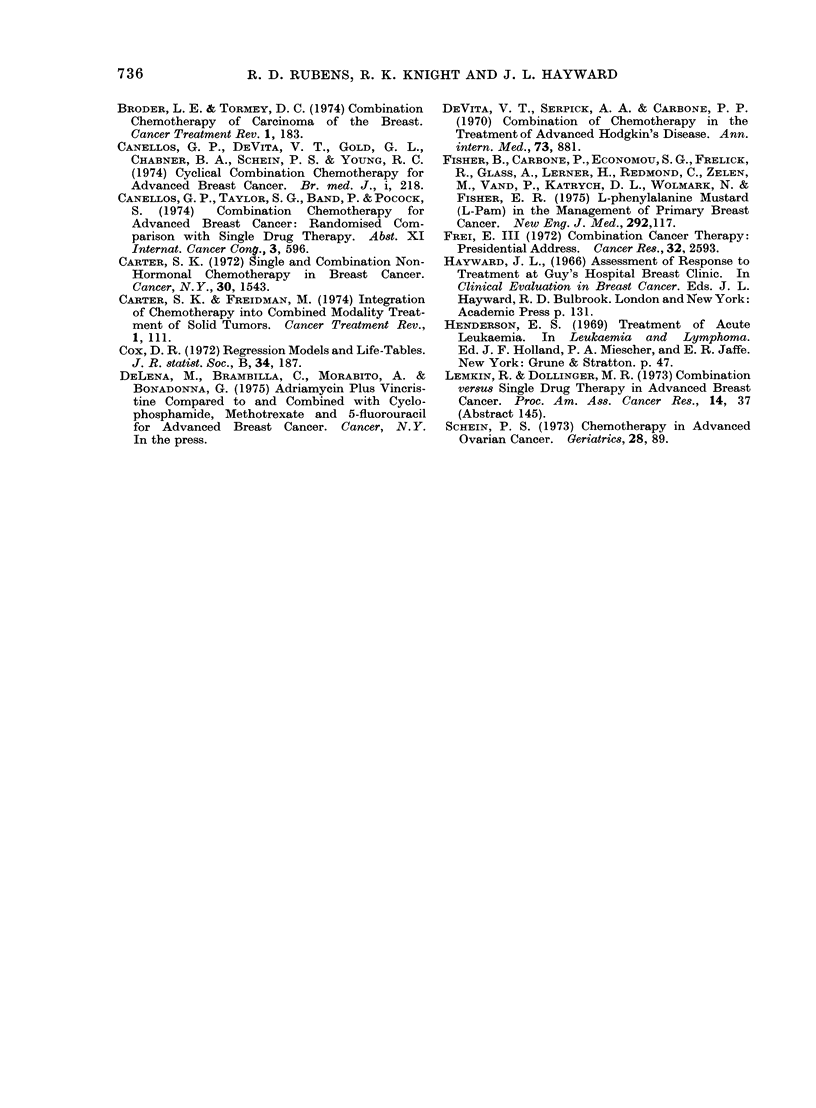# Chemotherapy of advanced breast cancer: a controlled randomized trial of cyclophosphamide versus a four-drug combination.

**DOI:** 10.1038/bjc.1975.284

**Published:** 1975-12

**Authors:** R. D. Rubens, R. K. Knight, J. L. Hayward

## Abstract

Ninety-nine patients with advanced breast cancer were randomized to receive either cyclophosphamide continuously or a combination of cyclophosphamide, methotrexate, 5-fluorouracil and vinblastine given intermittently. The number and duration of objective responses were greater in patients receiving the combination but the differences between the two treatments did not achieve formal significance. The combination was logistically easier to manage and produced less toxicity.


*Br. J. Cancer* (1975) **32**, 730

### CHEMOTHERAPY OF ADVANCED BREAST CANCER: A CONTROLLED RANDOMIZED TRIAL OF CYCLOPHOSPHAMIDE *VERSUS* A FOUR-DRUG COMBINATION

R. D. RUBENS, R. K. KNIGHT AND J. L. HAYWARD

*From the Imperial Cancer Research Fund, Breast Cancer Unit, Guy's Hospital, London SE1 9RT*

Received 22 July 1975. Accepted 22 August 1975

**Summary.—Ninety-nine patients with advanced breast cancer were randomized to receive either cyclophosphamide continuously or a combination of cyclophosphamide, methotrexate, 5-fluorouracil and vinblastine given intermittently. The number and duration of objective responses were greater in patients receiving the combination but the differences between the two treatments did not achieve formal significance. The combination was logistically easier to manage and produced less toxicity.**

IN RECENT years, there have been some notable advances in the results of cancer chemotherapy by using drugs in combination instead of singly (Frei, 1972). Major improvements have occurred in the treatment of acute lymphoblastic leukaemia (Henderson, 1969) and some lymphomata, particularly advanced Hodgkin's disease (DeVita, Serpick and Carbone, 1970). The common carcinomata are less responsive and, in gastrointestinal (Carter and Friedman, 1974) and ovarian cancer (Schein, 1973), combinations of drugs are not superior to single agents. It is now believed that in advanced breast cancer combination chemotherapy produces more remissions which are of longer duration than those obtained with the agents used singly. The evidence for this has recently been reviewed (Broder and Tormey, 1974) but it has not been formally established in a controlled trial. Such a study was started at the Breast Unit, Guy's Hospital in June 1970 and the results to the end of December 1974 are now reported.

### PATIENTS AND METHODS

Ninety-nine patients with advanced breast cancer entered the trial. None had had previous chemotherapy and all had relapsed after, or had failed to respond to, previous endocrine therapy. There were 4 categories of patients: (1) those who had had a hypophysectomy but had failed to respond or had subsequently relapsed; (2) those considered too old for hypophysectomy (over 65 years); (3) those selected as unlikely to benefit from hypophysectomy because they had had a period free from disease after excision of the primary tumour of less than 2 years, or were less than 6 years post-menopausal, except in cases where discriminant (Atkins *et al.*, 1968) was positive; (4) those considered medically unfit for hypophysectomy.

The patients were randomly allocated to one of 2 treatment groups, namely cyclophosphamide alone (C) or a regimen of 4 drugs (4D). Group C were treated as had been the usual practice in this Unit for about 10 years. They received cyclophosphamide orally at a daily dose depending on body weight (<48 kg, 200 mg; 48–58 kg, 250 mg; >58 kg, 300 mg). The white blood cell count was estimated weekly and treatment was stopped if this fell below $2000/\mu l$; it was resumed when it had risen to $3000/\mu l$.

The selection of the 4 drugs was determined by the known efficiency of cyclophosphamide. 5-fluorouracil, methotrexate and the vinca-alkaloids in breast cancer (Carter, 1972). Vinblastine was chosen rather than vincristine (which has been used more often) in order to avoid neurotoxicity during an anticipated long period of treatment.

Group 4D received courses of treatment as follows:

Day 1 Oral cyclophosphamide 100 mg daily started and continued for 14 days.

Intravenous infusion of 300 ml saline

| | | |
|---|---|---|
| 5-fluorouracil | 500 mg | Injected separately into infusion tubing |
| Methotrexate | 25 mg | |
| Vinblastine | 5 mg | |

Day 8 Infusion and intravenous drugs as Day 1

Day 15 Infusion and intravenous drugs as Days 1 and 8
Cyclophosphamide stopped

After a 4-week rest period the cycle, starting at Day 1, was repeated. A course was started only if the white blood cell count was above 3000/$\mu$l. The second and third infusions (Days 8 and 15) were postponed if the white blood cell count fell below 2000, when the cyclophosphamide was also stopped.

In both Groups C and 4D treatment was continued indefinitely but was stopped if the disease progressed or if the patient relapsed after a successful response. At the beginning of treatment patients were assessed by physical examination, chest x-ray and skeletal survey. All visible and palpable lesions (including liver enlargement) were measured and/or photographed. These records provided a baseline against which to assess the response to chemotherapy. Group C patients were seen at 4-weekly intervals while Group 4D patients were assessed one week before each course of treatment.

*Assessment*

The following methods of assessment were used:

*Objective response.*—This means a measurable decrease in the size of one or more evaluable lesions or sclerosis of lytic bone metastases not accompanied by concurrent increase in other lesions nor the appearance of new lesions. Disappearance of all known lesions was deemed a "complete remission". The duration of a response is the time from the start of chemotherapy to the time of relapse or the appearance of new lesions. Although "objective response" as defined in this way is not believed to be a good index of the overall benefit from treatment, it is a criterion frequently used in the literature and has been used here so that the results described in this paper can be compared with other published work.

*Mean clinical value* (*MCV*).—This method of clinical assessment has been previously described in detail (Hayward, 1966). Briefly, all evaluable lesions are compared with baseline measurements periodically (usually 4-weekly) during treatment. For a given lesion a score of 1 denotes no change, 2 denotes improvement and 0 deterioration; new lesions score 0. After grouping the lesions into systems, a score for each system is obtained by dividing the sum of the scores by the number of lesions and multiplying by 6. The scores for each system are then averaged to give the mean clinical value (MCV). The maximum possible MCV is 12 which means all lesions are in regression, while an MCV of 6 (which is the baseline score) indicates no overall change; values below 6 are obtained when the disease progresses despite treatment and reach 0 when all lesions have measurably deteriorated. A comparison of the responses to different treatments can be made from the MCV at a given time from the start of treatment (3 months in this study) or from the total MCV which is the sum of the MCVs at 4-weekly intervals for the duration of therapy.

*Success rate.*—Treatment was considered successful, intermediate or a failure over a defined time period (3, 6, 12 and 24 months in this study) using the criteria of the British Breast Group (1974).

*Success* means a measurable improvement of all known lesions persisting at the end of the defined time period during which no new lesions should have appeared. *Intermediate* includes all responses which do not last the defined period and those cases in which some lesions regress while others get worse, or when lesions remain static. *Failure* indicates worsening of the disease for the duration of treatment.

*Survival.*—Survival from the start of chemotherapy has been analysed by the life-table method and by the method of Cox (1972).

*Statistical methods* (Armitage, 1971).—Differences between the C and 4D groups were compared by the $t$ test for duration of response and MCV; in addition, Willcoxon's rank sum test was used for MCV. Success rates were compared using the chi-square test.

### RESULTS

Forty-nine patients were randomly allocated to Group C and 50 to Group 4D; no patient was excluded from the analysis. Although 3 of the 4D patients died in the short interval between randomization and before starting chemotherapy they were still included in the analysis. This was done to maintain strict comparability between the two groups, particularly because the Group C patients were able to start treatment without delay whereas Group 4D usually waited for a few days for the first course of intravenous therapy. The characteristics of the two groups with regard to age (Table I), time from diagnosis to start of chemotherapy (Table II), previous treatment (Table III) and extent of disease at the start of chemotherapy (Table IV) were similar.

All patients have been followed up for a minimum of 6 months from the start of chemotherapy. At the time of analysis only 6 patients were still in remission in Group C and 5 in Group 4D. Four patients in each group were alive on subsequent therapy having failed on, or relapsed after, chemotherapy. The remaining 80 patients have died.

*Objective responses* (Table V)

There were more objective responses in Group 4D and the duration of these was longer but the difference between the two treatments was not statistically significant. The response rates according to anatomical sites are shown in Table IV. Treatment was most effective against lesions in the breast, lymph nodes and skin, particularly in Group 4D. Skeletal lesions appeared to respond poorly but this is probably due to the long time needed for sclerosis of lytic lesions to become detectable on radiographs. Hepatic metastases assessed by liver size regressed in about half of

TABLE I.—*Age at Diagnosis*

| | Numbers of Patients | |
|---|---|---|
| Years | Group C | Group 4D |
| 21–30 | 1 | 0 |
| 31–40 | 7 | 7 |
| 41–50 | 14 | 16 |
| 51–60 | 15 | 16 |
| 61–70 | 10 | 8 |
| 71–80 | 2 | 3 |

TABLE II.—*Time from Diagnosis to Start of Chemotherapy*

| | Numbers of Patients | |
|---|---|---|
| Months | Group C | Group 4D |
| 0–12 | 5 | 6 |
| 13–24 | 14 | 12 |
| 25–60 | 19 | 21 |
| $>60$ | 11 | 11 |

TABLE III.—*Previous Treatment*

| | Numbers of Patients | |
|---|---|---|
| | Group C | Group 4D |
| Surgical excision of primary tumour (wide excision, simple mastectomy or radical mastectomy) ± radiotherapy | 30 | 32 |
| Oophorectomy | 15 | 15 |
| Androgens | 19 | 23 |
| Oestrogens | 20 | 16 |
| Hypophysectomy or Adrenalectomy | 28 | 30 |
| Prednisone | 7 | 11 |

TABLE IV.—*Sites of Involvement and Response*

| | No. of patients | |
|---|---|---|
| | Group C | Group 4D |
| Breast | 9/21 (43%) | 16/23 (70%) |
| Lymph Nodes | 18/33 (55%) | 22/33 (67%) |
| Skin | 17/33 (52%) | 21/32 (66%) |
| Skeleton | 7/26 (27%) | 6/21 (29%) |
| Lungs | 2/9 | 3/5 |
| Pleura | 1/14 | 3/13 |
| Liver | 8/14 | 5/11 |
| Ascites | 2/5 | 1/4 |
| Abdominal mass | 0/2 | – |
| Brain | – | 0/1 |

* Denominators indicate the number of patients with involvement of the stated site at the start of chemotherapy and numerators show the number with a response at that site.

TABLE V.—*Objective Responses*

| | | No. of patients | | |
|---|---|---|---|---|
| | | Group C | Group 4D | |
| No. of patients | | 49 | 50 | |
| Objective responses | | 27 (55%) | 31 (62%) | |
| Complete remissions | | 2 | 1 | |
| Duration of response (months) | | | | |
| All patients: | Mean | 3·9 | 5·5 | $P<0·2$ |
| | Median | 2 | 3 | |
| | Range | 0–24 | 0–30 | |
| Objective responders only: | Mean | 7·1 | 8·8 | $P<0·3$ |
| | Median | 5·5 | 7 | |
| | Range | 2–24 | 1–30 | |

TABLE VI.—*Mean Clinical Value*

| | | No. of patients | | |
|---|---|---|---|---|
| | | Group C | Group 4D | |
| MCV at 3 months | All patients | 4·0 | 5·7 | $P<0\cdot1$ |
| | Objective responders only | 7·5 | 8·9 | $P<0\cdot2$ |
| Total MCV | All patients | 42·0 | 61·0 | $P<0\cdot2$ |
| | Objective responders only | 75·0 | 96·0 | $P<0\cdot3$ |

TABLE VII.—*Success Rate*

| | | Success | Intermediate | Failure | No. of patients evaluable at time stated |
|---|---|---|---|---|---|
| 3 Months | C | 16 (33%) | 11 | 22 | 49 |
| | 4D | 26 (52%) | 5 | 19 | 50 |
| 6 Months | C | 13 (26%) | 14 | 22 | 49 |
| | 4D | 19 (38%) | 12 | 19 | 50 |
| 12 Months | C | 4 (9%) | 20 | 22 | 46 |
| | 4D | 7 (15%) | 21 | 19 | 47 |
| 24 Months | C | 1 | 21 | 22 | 44 |
| | 4D | 1 | 25 | 19 | 45 |

patients in each group with involvement at this site, but other visceral lesions were less responsive.

*Mean clinical value*

The MCV at 3 months and the total MCV averaged for each group are compared in Table VI. Although all the values were greater in Group 4D, the difference was not statistically significant with either of the two tests of significance used.

*Success rate*

There were more successes at 3, 6 and 12 months in Group 4D (Table VII) but this was not statistically significant ($P<0\cdot1$ at 3 months). At 24 months there was only one success in each group.

*Survival*

This was similar for both groups of patients (Fig). Analysis by the method of Cox (1972) showed that the difference in survival was not significant. After correction for the slight initial disparity in the involvement of different sites, which favoured Group 4D, the two groups are estimated to have had death rates in the ratio 1·15 (for C) to 1 (for 4D); 95% confidence limits for this ratio are 0·73 and 1·82. It is appropriate to note that after relapse 12 patients in Group C then received the 4 drug combination less cyclophosphamide. In addition, some patients in both groups received other treatments on relapse, such as norethisterone acetate, prednisone, adriamycin or radiotherapy (Table VIII).

*Modification to treatment due to toxicity*

In Group C the cyclophosphamide treatment was interrupted and/or the dose reduced in 31 patients because of leukopenia (20 patients), thrombocytopenia (1 patient), mouth ulcers (1 patient) or nausea and vomiting (6 patients). Four patients went on to intravenous

TABLE VIII.—*Subsequent Therapy Received by Patients after Failing or Relapsing on the Trial*

| | No. of patients | |
|---|---|---|
| | Group C | Group 4D |
| Norethisterone acetate | 4 | 9 |
| Prednisone | 1 | 1 |
| Hypophysectomy | 1 | 0 |
| Radiotherapy | 2 | 2 |
| 4D less cyclophosphamide | 12 | – |
| Adriamycin | 1 | 4 |

FIG.—Survival curves calculated by the life-table method (– – – – Group C; ———— Group 4D).

cyclophosphamide because of intolerable nausea and vomiting. In one patient cyclophosphamide was withdrawn when she developed severe non-thrombocytopenic purpura, although this was probably the result of the administration of an analgesic drug. One patient in Group C developed irreversible marrow aplasia and died from a septicaemia. Two patients had local radiotherapy to lesions during chemotherapy.

In Group 4D the second or third intravenous doses in a course were occasionally omitted in 15 patients because of leukopenia; this was a frequent occurence in only 2 patients, one of whom eventually continued treatment with cyclophosphamide only. Methotrexate was ultimately omitted in one patient and 5-fluorouracil in another because of mouth ulcers, whilst in a further patient this side-effect was controlled by a reduction in the dose of 5-fluorouracil. In one patient cyclophosphamide was withdrawn after course 2 because of unacceptable nausea and vomiting. In one patient vincristine was substituted for vinblastine and cyclophosphamide stopped after course 5 in order to prevent further severe leukopenia which had been responsible for a septicaemic episode. One patient required the insertion of an A-V shunt after the 4th course in order that the intravenous drugs could be continued. One patient in this group had a septicaemia during a period of leukopenia but this was treated successfully and chemotherapy was resumed. Irreversible marrow aplasia did not occur in any patient on the 4-drug combination.

### DISCUSSION

The value of chemotherapy in advanced breast cancer is established and responders to chemotherapy have been shown to have a longer survival than non-responders (Baker *et al.*, 1974; Canellos *et al.*, 1974*a*). Many reports have suggested that multiple drugs, in combination, given intermittently are more effective than the agents used singly (Broder and Tormey, 1974), but this has not been shown unequivocally in a clinical trial. In a small randomized trial, 5-fluorouracil was shown to be as effective as a 5-drug combination of 5-fluorouracil, methotrexate, vincristine, prednisone and chlorambucil, both with regard to response rate and duration of response (Lemkin and Dollinger, 1973). The Eastern Co-Operative Oncology Group has demonstrated clearly the superiority of a combination of cyclophosphamide, methotrexate and 5-fluorouracil over L-phenylalanine mustard used singly (Canellos *et al.*, 1974*b*). However, the response rate of 20% to L-phenylalanine mustard in this trial was inferior to the responses expected from any of the other agents if they had been used singly (Carter, 1972), and so the trial did not prove that combinations are superior to single agents.

The results of the present study suggests that a combination of cyclophosphamide, methotrexate, 5-fluorouracil and vinblastine may result in a greater number of responses, which last for a longer time than cyclophosphamide alone although the difference between the treatments does not achieve formal significance.

Survival in the two groups was the same but this was expected as other treatments were available to relapsed patients. Moreover, patients in Group C who failed to respond were then given combination chemotherapy. Administration of the 4-drug combination was easier to control than continuous cyclophosphamide and it produced less toxicity, particularly with regard to cystitis and serious marrow suppression.

It would seem then that, although the use of drug combinations probably represents an advance in the treatment of breast cancer, this has not been proved in the present trial. However, combination chemotherapy is still in its infancy and further improvements are likely. The single most effective drug now in breast cancer is adriamycin (Blum and Carter, 1974) and the use of this agent in combination with other drugs may achieve a greater degree of benefit (Brambilla, DeLena and Bonadonna, 1974; DeLena *et al.*, 1975). The ultimate role of chemotherapy in breast cancer may be at an earlier stage of the disease when the eradication of all malignant cells might be possible (Fisher *et al.*, 1975).

We are grateful to Professor P. Armitage for his advice in the analysis of these results.

### REFERENCES

ARMITAGE, P., (1971) *Statistical Methods in Medical Research.* Oxford and Edinburgh: Blackwell Scientific Publications.

ATKINS, H., BULBROOK, R. D., FALCONER, M. A., HAYWARD, J. L., MACLEAN, K. S. & SCHURR, P. H. (1968) Ten Years' Experience of Steroid Assays in the Management of Breast Cancer. *Lancet*, ii, 1255.

BAKER, L. H., VAUGHN, C. B., AL-SARRAF, M., REED, M. L. & VAITKEVICIUS, V. K. (1974) Evaluation of Combination *versus* Sequential Cytotoxic Chemotherapy in the Treatment of Advanced Breast Cancer. *Cancer, N.Y.*, **33**, 513.

BLUM, R. H. & CARTER, S. K. (1974) Adriamycin. *Ann. intern. Med.*, **80**, 249.

BRAMBILLA, C., DELENA, M. & BONADONNA, G., (1974) Combination Chemotherapy with Adriamycin (NSC–123127) in Metastatic Mammary Carcinoma. *Cancer Chemother. Rep.* Part 1, **58**, 251.

BRITISH BREAST GROUP (1974) Assessment of Response to Treatment in Advanced Breast Cancer. *Lancet*, ii, 38.

BRODER, L. E. & TORMEY, D. C. (1974) Combination Chemotherapy of Carcinoma of the Breast. *Cancer Treatment Rev.* **1**, 183.

CANELLOS, G. P., DEVITA, V. T., GOLD, G. L., CHABNER, B. A., SCHEIN, P. S. & YOUNG, R. C. (1974) Cyclical Combination Chemotherapy for Advanced Breast Cancer. *Br. med. J.*, i, 218.

CANELLOS, G. P., TAYLOR, S. G., BAND, P. & POCOCK, S. (1974) Combination Chemotherapy for Advanced Breast Cancer: Randomised Comparison with Single Drug Therapy. *Abst.* XI *Internat. Cancer Cong.*, **3**, 596.

CARTER, S. K. (1972) Single and Combination Non-Hormonal Chemotherapy in Breast Cancer. *Cancer, N.Y.*, **30**, 1543.

CARTER, S. K. & FREIDMAN, M. (1974) Integration of Chemotherapy into Combined Modality Treatment of Solid Tumors. *Cancer Treatment Rev.*, **1**, 111.

COX, D. R. (1972) Regression Models and Life-Tables. *J. R. statist. Soc.*, B, **34**, 187.

DELENA, M., BRAMBILLA, C., MORABITO, A. & BONADONNA, G. (1975) Adriamycin Plus Vincristine Compared to and Combined with Cyclophosphamide, Methotrexate and 5-fluorouracil for Advanced Breast Cancer. *Cancer, N.Y.* In the press.

DEVITA, V. T., SERPICK, A. A. & CARBONE, P. P. (1970) Combination of Chemotherapy in the Treatment of Advanced Hodgkin's Disease. *Ann. intern. Med.*, **73**, 881.

FISHER, B., CARBONE, P., ECONOMOU, S. G., FRELICK, R., GLASS, A., LERNER, H., REDMOND, C., ZELEN, M., VAND, P., KATRYCH, D. L., WOLMARK, N. & FISHER, E. R. (1975) L-phenylalanine Mustard (L-Pam) in the Management of Primary Breast Cancer. *New Eng. J. Med.*, **292**,117.

FREI, E. III (1972) Combination Cancer Therapy: Presidential Address. *Cancer Res.*, **32**, 2593.

HAYWARD, J. L., (1966) Assessment of Response to Treatment at Guy's Hospital Breast Clinic. In *Clinical Evaluation in Breast Cancer*. Eds. J. L. Hayward, R. D. Bulbrook. London and New York: Academic Press p. 131.

HENDERSON, E. S. (1969) Treatment of Acute Leukaemia. In *Leukaemia and Lymphoma*. Ed. J. F. Holland, P. A. Miescher, and E. R. Jaffe. New York: Grune & Stratton. p. 47.

LEMKIN, R. & DOLLINGER, M. R. (1973) Combination *versus* Single Drug Therapy in Advanced Breast Cancer. *Proc. Am. Ass. Cancer Res.*, **14**, 37 (Abstract 145).

SCHEIN, P. S. (1973) Chemotherapy in Advanced Ovarian Cancer. *Geriatrics*, **28**, 89.